# Evaluation of Virulence Determinants Using Whole-Genome Sequencing and Phenotypic Biofilm Analysis of Outbreak-Linked *Staphylococcus aureus* Isolates

**DOI:** 10.3389/fmicb.2021.687625

**Published:** 2021-06-29

**Authors:** Jennifer M. Hait, Guojie Cao, George Kastanis, Lanlan Yin, James B. Pettengill, Sandra M. Tallent

**Affiliations:** ^1^Division of Microbiology, Center for Food Safety and Applied Nutrition, U.S. Food and Drug Administration, Office of Regulatory Science, College Park, MD, United States; ^2^Center for Food Safety and Applied Nutrition, U.S. Food and Drug Administration, Office of Analytics and Outreach, College Park, MD, United States

**Keywords:** staphylococcal food poisoning, staphylococcal enterotoxins, WGS, biofilm, *Staphylococcus aureus*

## Abstract

Biofilms are a frequent cause of food contamination of potentially pathogenic bacteria, such as *Staphylococcus aureus*. Given its vast role in human disease, the possible impact of biofilm-producing *S. aureus* isolates in a food processing environment is evident. Sixty-nine *S. aureus* isolates collected from one firm following multiple staphylococcal food poisoning outbreak investigations were utilized for this analysis. Strain evaluations were performed to establish virulence determinants and the evolutionary relationships using data generated by shotgun whole-genome sequencing (WGS), along with end point polymerase chain reaction (PCR) and *in vitro* phenotypic assessments. *S. aureus* isolates were grouped into six well-supported clades in the phylogenetic tree, with the relationships within the clades indicating a strong degree of clonal structure. Our analysis identified four major sequence types 47.8% ST1, 31.9% ST45, 7.2% ST5, and 7.2% ST30 and two major *spa* types 47.8% t127 and 29.0% t3783. Extrapolated staphylococcal enterotoxin (SE) analysis found that all isolates were positive for at least 1 of the 23 SEs and/or SE-like toxin genes. Enterotoxigenic assessments found that 93% of the isolates expressed a classical SE(A–E). SE gene concurrence was observed at 96.2%, based on PCR and WGS results. In total, 46 gene targets were distinguished. This included genes that encode for adhesion and biofilm synthesis such as *clfA*, *clfB*, *bbp*, *ebpS*, *ica*, *bap* and *agr*. Our evaluation found *agr* group III to be the most prevalent at 55%, followed by 35% for *agr* group I. All isolates harbored the complete intercellular adhesion operon that is recognized to contain genes responsible for the adhesion step of biofilm formation by encoding proteins involved in the syntheses of the biofilm matrix. Phenotypic characterization of biofilm formation was evaluated three times, with each test completed in triplicate and accomplished utilizing the microtiter plate method and Congo red agar (CRA). The microtiter plate results indicated moderate to high biofilm formation for 96% of the isolates, with 4% exhibiting weak to no biofilm development. CRA results yielded all positive to intermediate results. The potential to inadvertently transfer pathogenic bacteria from the environment into food products creates challenges to any firm and may result in adulterated food.

## Introduction

*Staphylococcus aureus* is the known cause of many diseases and is considered one of the most significant pathogens for humans. It is a frequent source of infections, such as pneumonia, postoperative wound infections, endocarditis, and bacteremia. The secreted toxins and cell wall-associated virulence factors contribute to the bacterium’s ability to adhere to, proliferate in, and potentially destroy host cells and tissue matrix (Seo and Bohach, 2007). Enterotoxigenic *Staphylococcus* strains are also a common cause of staphylococcal food poisoning (SFP) that affects an estimated 241,148 individuals the United States annually (Scallan et al., 2011).

*Staphylococcus* species are ubiquitous in the environment and is often found in the air, dust, water, and food, and exist on humans and animals (Hennekinne et al., 2010). *S. aureus* and *S. epidermidis* are commensal inhabitants of the human skin and consequently have increased probability to be present in foods handled directly by humans and on food contact surfaces. Inadequate hygiene practices in food processing plants may result in contamination of food products with an enterotoxin-producing strain of *S. aureus* (Hait et al., 2014). Biofilms are a frequent source of food contamination of potentially pathogenic bacteria, such as *S. aureus*. Biofilms are the most common bacterial lifestyle in nature, and as the cells multiply, they secrete a matrix of extracellular polymeric substances (EPS) that wraps the cells (Gutiérrez et al., 2012). The most commonly found bacteria on food contact surfaces in dairy environments are *Enterobacter*, *Lactobacillus*, *Listeria*, *Micrococcus*, *Streptococcus*, *Staphylococcus*, *Bacillus*, and *Pseudomonas* (Gutiérrez et al., 2012). The presence of biofilm-forming strains of *S. aureus* in food processing environments and in food are equally important as biofilms are involved in cross-contamination events.

The two-step process in biofilm development first involves the bacteria adherence to a surface followed by the bacteria multiplying to form a multilayered biofilm associated with the production of polysaccharide intercellular adhesion (PIA) (Nasr et al., 2012). PIA is produced and secreted by the proteins encoded in the intercellular adhesion (*icaABCD*) operon (Miao et al., 2019). *S. aureus* adherence is facilitated by protein adhesions of the microbial surface components recognizing adhesive matrix molecules (MSCRAMMs), which is most frequently anchored to the cell wall peptidoglycan (Foster and Höök, 1998). *S. aureus* genes that encode MSCRAMMs include bone sialoprotein binding protein (*bbp*), collagen binding protein (*cna*), elastin binding protein (*ebpS*), encoding laminin binding protein (*eno*), encoding fibrinogen binding protein (*fib*), fibronectin binding proteins A (*fnbA*), fibronectin binding proteins A (*fnbB*), clumping factors A (*clfA*), and clumping factors B (*clfB*) genes (Tristan et al., 2003). The *icaABCD* operon is responsible for the slime production in *S. aureus* and *S. epidermidis* (Cucarella et al., 2001). This operon contains *ica* genes, responsible in the *icaABCD* step of biofilm formation by encoding proteins involved in the syntheses of the biofilm matrix polysaccharide–poly-*N*-succinyl β-1-6 glucosamine (PIA–PNSG) (Cucarella et al., 2001). The *icaA* and *icaD* genes are known to play a significant role in biofilm formation. The *icaA* gene encodes *N*-acetylglucosaminyltransferase, an enzyme involved in PIA synthesis, and the *icaD* gene has been described as critical in the maximal expression of *N*-acetylglucosaminyltransferase resulting in complete phenotypic expression of the capsular polysaccharide (Nasr et al., 2012). The DtlA and Bap proteins have been recognized to influence biofilm formation for *S. aureus*. The staphylococcal accessory regulator (SarA) is an essential element that controls production of *S. aureus* virulence factors and was found to be vital in a study for biofilm development (Valle et al., 2003). The accessory gene regulator (*agr*) quorum-sensing system has been described as central to the role in *S. aureus* pathogenesis (Yarwood and Schlievert, 2003). Biofilm synthesis is mainly encoded by *ica*, *bap*, and *agr* genes, with *clfA*, *clfB*, *bbp*, and *ebpS* genes also involved (Notcovich et al., 2018).

*Staphylococcus aureus* are capable of harboring a plethora of virulence factors. This includes the staphylococcal enterotoxin (SE) superfamily of secreted virulence factors that share both functional and structural similarities (Fisher et al., 2018). There are SEs and SE-like toxins (SEI); SEA, SEB, SEC_1,2,3_, SED, SEE, SEG, SEH, SEI, SE*l*J, SEK, SEL, SEM, SEN, SEO, SEP, SEQ, SER, SES, SET, SE*l*U, SE*l*W SE*l*V, SE*l*X, and SE*l*Y (Fisher et al., 2018). The SE group is recognized to exhibit emetic activity, while the SEl toxins have not been established to cause emesis or gastroenteritis in primates (Fisher et al., 2018). For example, previous analysis determined SE*l*J to be non-emetic, while SE*l*U, SE*l*V, SE*l*W, SE*l*X, and SE*l*Y have not been tested in non-human primates (Fisher et al., 2018). Using a comparison of the nucleotide and amino acid sequences, SEs and SEls are grouped into four evolutionary groups: SEA group 1 (SEA, SED, SEE, SElJ, SEH, SEN, SEO, SEP, and SES), SEB group 2 (SEB, SECs, SEG, SER, SElU, and SElW), SEI group 3 (SEI, SEK, SEL, SEQ, SEM, and SElV), and SElX group 4 (TSST-1, SET, SElX, and SElY) (Fisher et al., 2018). Overall, these enterotoxin and enterotoxin-like proteins are described as single-chain proteins with molecular weights ranging from 22 to 29 kDa and are known to exhibit superantigenic activity resulting in non-specific T-cell proliferation (Hennekinne et al., 2010). These toxins can be encoded in plasmids, prophages, and on chromosomal pathogenicity islands (Rajkovic et al., 2019). SE genes on mobile genetic elements create an additional risk factor for transmission due to the potential of horizonal gene transfer (Rajkovic et al., 2019).

Ingestion of SEs produced by some *S. aureus* strains are the etiological agent of common illness called SFP. The extracellular SEs are resistant to various environmental conditions and are resistant to proteolytic enzymes such as trypsin and pepsin meaning the SEs persist in the digestive tract after ingestion (Hait et al., 2014). SFP has a rapid onset of 1–7 h following ingestion. The SE intoxication results in nausea, emesis, abdominal cramping, and diarrhea that typically last less than 24–48 h (Hennekinne et al., 2010).

In this study, an evaluation between two tests for phenotypic biofilm formation and the correlation of the prevalence of genes involved in biofilm production/formation were investigated for 69 *S. aureus* isolates from outbreak investigations. The genomic sequencing tools were used to identify virulence gene targets in these outbreak-linked *S. aureus* isolates. A total of 601 environmental swabs and ingredient samples were collected and analyzed, in which 14% were confirmed as *S. aureus*. Whole-genome sequencing (WGS) and biofilm analysis were conducted on 69 of these *S. aureus* isolates. The isolates were collected on three dates, the initial investigation date December 27, 2010, the post-clean up investigation date January 10, 2011, and the final investigation date November 18, 2011. The collection locations or sources in the bakery include assembly production room, baking production room, final product storage room, ingredients storage room, and products.

This study aimed to investigate the phylogenetic relationships and virulence determinants of 69 outbreak-linked environmental and food acquired *S. aureus* isolates. *S. aureus* phenotypes such as adhesion and biofilm formation are important since they promote the colonization of food environments (Gutiérrez et al., 2012). All 69 isolates were screened for the genes associated with biofilm formation *bap*, *icaA*, and *icaD*, and for genes that encode MSCRAMMs *bbp*, *cna*, *ebpS*, *eno*, *fib*, *fnbA*, *fnbB*, *clfA*, and *clfB* genes. Sequence-based analysis was used to identify 23 SE genes as well as additional virulence factors such as *pvl* (Panton–Valentine leukocidin). PVL is a cytotoxin composed of two components, LukS-PV and LukF-PV. These secreted components cause neutrophil lysis after assembling into a pore-forming heptamer on neutrophil membranes (Shallcross et al., 2013). In total, 46 genes were investigated. Phenotype testing was accomplished to demonstrate biofilm formation capabilities and the expression of SEs SEA–SEE.

## Materials and Methods

The outbreak isolates of *S. aureus* (*n* = 69) used in this study are maintained in the strain collection of FDA’s Center for Food Safety and Applied Nutrition. These isolates were confirmed phenotypically using Baird Parker + Rabbit Plasma Fibrinogen (RPF) agar as the selective plating (Item number: 43531, bioMérieux, Marcy-l’Etoile, France) and biochemical testing using the VITEK^®^ 2 Identification of Gram-positive card (bioMérieux, Marcy-l’Etoile,France). Sixty-nine of the *S. aureus* isolates collected were assessed for genotype and phenotype virulence factors. These evaluations used a combination of end point polymerase chain reaction (PCR), WGS analysis, biofilm assays, and enterotoxin testing. The outbreak summaries previously published describe the PCR primers and method utilized for SE and SE-like gene identification and enterotoxigenic potential outcomes of these isolates (Hait et al., 2014). In this earlier research, each isolate was tested using the VIDAS Staph Enterotoxin II (bioMérieux, Marcy-l’Etoile, France) to determine the expression of SEA; SEB; SEC 1, 2, 3; SED; and SEE. The enterotoxigenic assessments established that 93% (64/69) of the isolates expressed a classical SE(A–E). The PCR results previously performed will be used to evaluate congruency with WGS results.

### Whole-Genome Sequencing Analysis

The genomic DNA was extracted using the DNAeasy Blood and Tissue Kit (Qiagen, Inc., Valencia, CA, United States) following an overnight grown culture incubated at 37°C in Trypticase Soy Broth (TSB) (Becton, Dickinson, Franklin Lakes, NJ, United States). The DNA concentration was measured using a Qubit 3.0 fluorometer (Life Technologies, Frederick, MD, United States). The libraries were prepared following the Illumina Nextera XT or Nextera Flex Library Prep protocols (Illumina, San Diego, CA, United States). The prepared libraries were sequenced on the Illumina MiSeq (Illumina, San Diego, CA, United States) using MiSeq Reagents Kits v2 (500 cycles) or MiSeq Reagents Kits v3 (600 cycles). Raw reads quality was assessed by the following parameters: cluster density (1200–1400 k/mm^2^) and percentage of clusters passing filters (>80%). Raw reads were trimmed using Trimmomatic (Bolger et al., 2014) and assembled *de novo* using SKESA v2.2 with minimal contig length as 500 bp (Souvorov et al., 2018). Annotations of assemblies were processed using the Prokka v1.14.5 (Seemann, 2014). Virulence genes were identified here using WGS data of these isolates (Hait et al., 2021). BLAST (Altschul et al., 1990)-based approach was used to identify presence–absence of enterotoxin genes SEA–SE*l*X, virulence factors, and biofilm-associated genes. kSNP 3.0 (Gardner et al., 2015) was used to generate the single-nucleotide polymorphism (SNP) matrix with the optimum k-mer size of 19. RAxML v8.2.9 (Stamatakis, 2014) was used to construct the maximum-likelihood phylogenetic tree (GTRGAMMA with 1000 rapid bootstrapping searches), and ggtree was used to visualize the phylogenetic tree (de Vienne et al., 2012; Wood and Salzberg, 2014; Yu et al., 2017). MLST 2.0 was used to identify the sequence types (Larsen et al., 2012) and spaTyper 1.0 was used to identify spa types (Bartels et al., 2014). GenBank accession numbers for the 69 *Staphylococcus aureus* isolates is provided in the Supplementary Table 1. In addition, we selected six *S. aureus* isolates from NCBI’s Pathogen Detection,^1^ which were isolated from the United States in recent years (SA1428_cattle_IA_2017, 1522_gallus_AR_2019, LS1_environment_WI_2019, CFSAN092696_rat feces_IL_2018, WAPHLSTAP00047_water_HI_2020, and WAPHLSTAP00089_ water_HI_2020, Figure 2) as reference in the phylogenetic analysis.

### Biofilm Testing

Biofilm analysis was conducted to determine the ability of each isolate to form a biofilm. This was accomplished using two previously described methods, the microtiter plate method and observations on Congo red agar (CRA) (Mathur et al., 2006; de Castro Melo et al., 2013). CRA was composed of 37 g/L of brain–heart infusion broth (Becton Dickinson, Franklin Lakes, NJ, United States), 50 g/L of sucrose (Sigma, St. Louis, MO, United States), 10 g/L of agar (Becton Dickinson, Franklin Lakes, NJ, United States), and 0.8 g/L of Congo red (Fisher Scientific, Waltham, MA, United States). Phenotypic characterization of biofilm formation of each isolate along with controls was evaluated three times, with each done in triplicate. American Type Culture Collection (ATCC, Manassas, VA, United States)-acquired control strains were utilized for biofilm determinations in the microtiter plate assay and to distinguish colonies on CRA. *S. epidermidis* ATCC 35984 is characterized as a high-level biofilm formation strain with black crystalline colonies on CRA, and *S. epidermidis* ATCC 12228 is a non-biofilm producer with pink colonies on CRA. Phenotypic biofilm examination using CRA identified biofilm producers as black colonies with dry crystalline consistency and non-biofilm-producing colonies developed smooth colonies that ranged from pink to red in color. Weak and moderate biofilm formers produced darkened brownish or black smooth colonies.

A microtiter plate assay was performed to test biofilm formation *in vitro* following a described method (de Castro Melo et al., 2013). Biofilm detection using the microtiter plate method used a sterile flat-bottom 96-well plate. These plates were inoculated with TSB (Becton Dickinson, Franklin Lakes, NJ, United States) containing 25% glucose and overnight cultures diluted at 1:100. These plates were incubated aerobically for 18–24 h at 37°C and then the wells were emptied followed by three consecutive washes using phosphate buffered saline solution, pH 7.2. The washed microtiter plates were dried for 1 h at 60°C and then stained with 1% crystal violet (Fisher Scientific, Waltham, MA, United States) for 1 min. The stained wells were washed three times with distilled water and allowed to dry at room temperature. Using a microplate reader, the optical density (OD) values measured at 490 nm were utilized as an index of surface bacteria adherence and biofilm formation. The uninoculated wells containing TSB with 25% glucose were the blanks used to correct the mean OD absorbance values for each *S. aureus* isolate. Based on the resulting absorbance values, the isolates were classified into three categories: positive result (high-level biofilm former) OD > 0.240, moderate result (intermediate-level biofilm former) OD = 0.240–0.120, and negative result (non-biofilm former) OD < 0.120.

## Results

**FIGURE 1:**
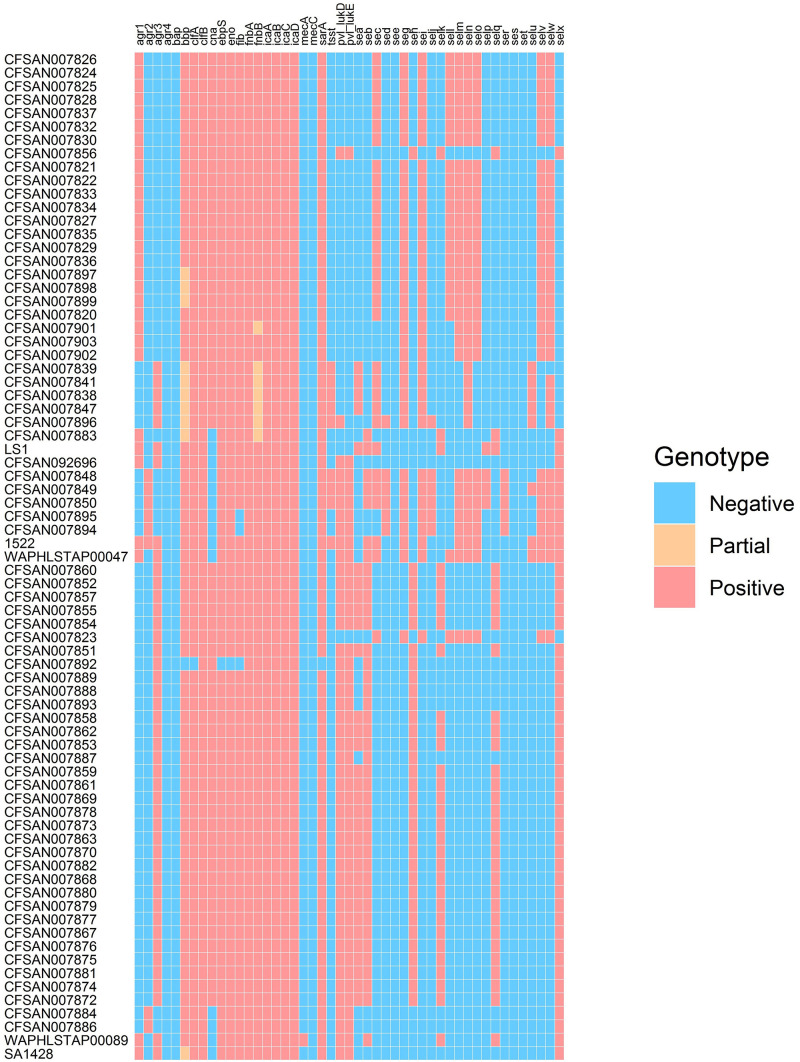
Heatmap illustrating genotype of outbreak-related *S. aureus* isolates.

The presence/absence of virulence factor encoding genes was determined for the outbreak-linked isolates using WGS analysis to identify 23 SE genes as well as additional virulence genes such as *pvl*, *tsst*, and biofilm-associated genes. These individual WGS results are illustrated in the heatmap exhibited in Figure 1. The overall prevalence of these virulence factor- and biofilm-associated genes is summarized in Table 1. The assessment of the *agr* group established that the *agr* group III was the most prevalent at 55%, followed by 35% for *agr* group I, and 10% for *agr* group II, while *agr* group IV was not present in the isolates tested. All of the isolates harbored the *icaABCD* genes *icaA*, *icaB*, *icaC*, and *icaD*. The clumping factor B gene, *clfB*, was detected in all of the isolates, while *clfA* was found in 99% of the outbreak isolates tested. None of the isolates harbored the biofilm-associated gene *bap*, while the genes encoding the *ebpS* and the *bbp* were present in all but one isolate, resulting in 99% prevalence. The incidence of additional genes that encode MSCRAMM was determined. The genes encoding fibronectin binding proteins A and B (*fnbA* and *fnbB*) were detected in all of the isolates, *fib* encoding fibrinogen binding protein were found in 96%, the *eno* gene was present in all but one isolate and *cna* encoding the collagen binding protein was found in 88% of the isolates.

**FIGURE 2:**
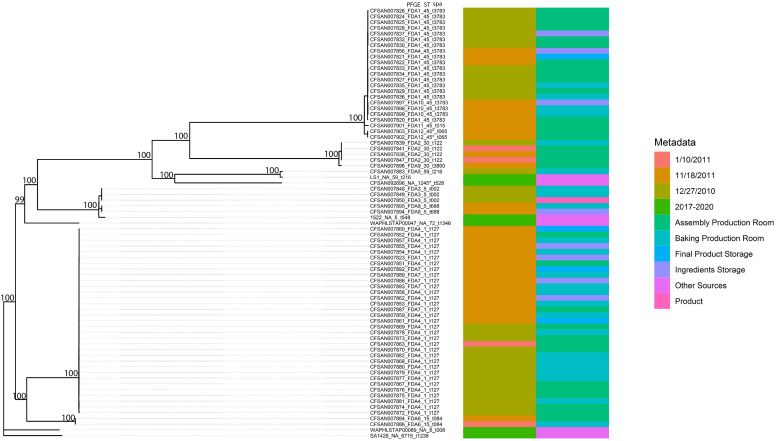
Phylogenetic tree inferred from a SNP matrix generated with kSNP3 and RAxML. Branch lengths are inferred number of substitutions per site and numbers at nodes represent percent bootstrap support.

**TABLE 1:** Incidence of virulence factors, including enterotoxin genes, for 69 outbreak-linked isolates using WGS analysis.

Gene	Encoding	Results (*n* = 69)
*agr1*	Accessory gene regulator I	35%
*agr2*	Accessory gene regulator II	10%
*agr3*	Accessory gene regulator III	55%
*agr4*	Accessory gene regulator IV	0
*bap*	Biofilm associated protein	0
*bbp*	Bone sialoprotein binding protein	99%
*clfA*	Clumping factor A	99%
*clfB*	Clumping factor B	100%
*cna*	Collagen binding protein	88%
*ebpS*	Elastin binding protein	99%
*eno*	Encoding laminin binding protein	99%
*fib*	Fibrinogen binding protein	96%
*fnbA*	Fibronectin binding protein A	100%
*fnbB*	Fibronectin binding protein B	100%
*icaA*	Intercellular adhesion A	100%
*icaB*	Intercellular adhesion B	100%
*icaC*	Intercellular adhesion C	100%
*icaD*	Intercellular adhesion D	100%
*mecA*	Regulates the expression of methicillin resistance	0
*mecC*	Regulates the expression of cefoxitin resistance	0
*sarA*	Staphylococcal accessory regulator	99%
*tsst*	Toxic shock syndrome toxin	12%
*pvl (_lukD)* and *(_lukE)*	Panton–Valentine leukocidin cytotoxin	58%
*sea*	Staphylococcal enterotoxin A	46%
*seb*	Staphylococcal enterotoxin B	54%
*sec*	Staphylococcal enterotoxin C	29%
*sed*	Staphylococcal enterotoxin D	9%
*see*	Staphylococcal enterotoxin E	0
*seg*	Staphylococcal enterotoxin G	48%
*seh*	Staphylococcal enterotoxin H	48%
*sei*	Staphylococcal enterotoxin I	48%
*selj*	Staphylococcal enterotoxin-like J	9%
*sek*	Staphylococcal enterotoxin K	42%
*sel*	Staphylococcal enterotoxin L	29%
*sem*	Staphylococcal enterotoxin M	46%
*sen*	Staphylococcal enterotoxin N	48%
*seo*	Staphylococcal enterotoxin O	41%
*sep*	Staphylococcal enterotoxin P	4%
*seq*	Staphylococcal enterotoxin Q	41%
*ser*	Staphylococcal enterotoxin R	7%
*ses*	Staphylococcal enterotoxin S	0
*set*	Staphylococcal enterotoxin T	0
*selu*	Staphylococcal enterotoxin-like U	9%
*selv*	Staphylococcal enterotoxin-like V	42%
*selw*	Staphylococcal enterotoxin-like W (formerly U2)	38%
*selx (alleles X9–X11)*	Staphylococcal enterotoxin-like X	58%

Examination for virulence determinant genes, such as *sarA*, *tsst*, *pvl*, *mecA*, and *mecC*, and SEs were also investigated using WGS during this study (Table 1). This investigation revealed that all except one of the isolates carried *sarA*. The gene encoding toxic shock syndrome toxin was present in 12% of the outbreak-associated isolates; 58% of the isolates carried the pvl gene, and *mecA* and *mecC* were not found in the 69 isolates. Genomic SE screening established that all isolates were positive for at least 1 of the 23 SEs and/or SEI genes. As illustrated in Table 1, the most abundant genes were *seb* and *selx*, found in greater than half of the isolates. The incidence of SE genes, *seg*, *seh*, *sei*, and *sen*, was observed at 48% and that of *sea* and *sem* was at 46%. The remaining SE genes were distinguished at a lesser frequency, with *see*, *ses*, and *set* genes absent in the isolates tested here. In Table 2, the congruency of each enterotoxin gene detected by both the PCR and WGS analysis was determined. Here, a higher number suggests a greater level of disagreement between the two assays.

**TABLE 2:** The congruence of enterotoxin genes detected by both the PCR assay and WGS analysis are expressed here as a percentage.

Gene	PCR only	WGS only
SEA	1.43	0
SEB	1.43	4.29
SEC	0	0
SED	0	1.43
SEE	0	0
SEH	0	0
SEI	0	0
SEJ	2.86	1.43
SEK	2.86	2.86
SEL	0	0
SEM	7.14	1.43
SEN	0	0
SEO	1.43	0
SEP	0	0
SEQ	0	40
SER	0	0
SEU	40	1.43
PVL (*lukD*)	0	4.29
PVL (*lukE*)	0	2.86

*A higher number suggests a greater incongruence between the two assays and the expected toxigenic phenotype.*

As demonstrated in Figure 2, the phylogenetic relationships among the 69 outbreak-related isolates and six reference genomes indicated a strong degree of population substructure where well-supported clades of nearly identical isolates were identified. In addition, in previous work (Hait et al., 2014), we identified 12 PFGE patterns among these isolates. The original 12 PFGE designations are exhibited in Figure 2, along with strain identifiers, the sample collection dates and locations, sequence type, and *spa* type. Most of the outbreak-related isolates belong to ST1 (33/69, 47.8%), ST45 (22/69, 31.9%), ST5 (5/69, 7.2%), and ST30 (5/69, 7.2%). Isolate CFSAN007883 belong to ST59 and CFSAN007902 and CFSAN007903 were new ST similar to ST45. In addition, we identified two major *spa* types: t127 (33/69, 47.8%) and t3783 (20/69, 29.0%). In this study, these isolates were grouped into six well-supported clades in the phylogenetic tree. Though some reference isolates from recent years (isolates 1522 and LS1) showed a close relationship with certain outbreak-related isolates, the majority of outbreak-related isolates in the current work presented clonal structure, such as the patterns FDA1 and FDA4 clades. Some PFGE patterns exclusively represent the isolates in some well-supported clades. For example, isolates CFSAN007848, CFSAN007849, and CFSAN007850 were collected on the same date, which all belong to Pattern FDA3 and shared the common ancestor by forming a cluster in the phylogenetic tree. This also applied to Patterns FDA2, FDA6, FDA8, FDA10, and FDA12. As expected, WGS data showed discrepancies compared to the PFGE results. Though most Pattern FDA1 isolates were grouped together with FDA10, FDA11, and FDA12 in the same well-supported clade, the FDA1 isolate CFSAN007823 was located in the clade mainly consisting of Patterns FDA4 and FDA7 isolates. Similarly, the FDA4 isolate CFSAN007856 was found in the clade mainly consisting of FDA1 isolates. In addition, we observed that closely related isolates were collected from different dates. The pattern FDA4 clade had isolates collected in December 2010, January 2011, and November 2011. This also applied to the pattern FDA2 cluster, which consisted of four isolates (CFSAN007838, CFSAN007839, CFSAN007841, and CFSAN007847) collected from the Assembly Production Room and Baking Production Room but different dates.

The microtiter plate assay used the mean blank-corrected absorbance values for each *S. aureus* isolate to classify them as positive (OD > 0.240), moderate (OD = 0.240–0.120), and negative (OD < 0.120) with regard to their ability to form a functional biofilm. This analysis indicated that 4.3% of the isolates examined resulted in weak to no biofilm formation, 36.2% were characterized as moderate, and 59.4% were high biofilm producers (Table 3). CRA examinations yielded 52.2% positive results identified by black colonies with dry crystalline consistency; 47.8% intermediate results differentiated by black/brownish smooth colonies and red smooth colonies characteristic of weak to non-biofilm producers were not identified in the isolates tested (Table 3).

**TABLE 3:** Genotypic identification of *agr* groups and *icaABCD* genes along with phenotypic classification of biofilm formation using CRA and microtiter plate assays for the *S. aureus* outbreak recovered isolates.

Strain identifier	Genotype		Phenotype		
		
	*agr* group type I–IV	*icaABCD* genes	CRA results	Mean OD values microtiter assay	Biofilm classification
CFSAN007820	I	Positive	Brown smooth	0.129	Moderate
CFSAN007821	I	Positive	Brown smooth	0.123	Moderate
CFSAN007822	I	Positive	Brown smooth	0.092	Negative
CFSAN007823	I	Positive	Brown smooth	0.149	Moderate
CFSAN007824	I	Positive	Brown smooth	0.14	Moderate
CFSAN007825	I	Positive	Brown smooth	0.209	Moderate
CFSAN007826	I	Positive	Brown smooth	0.101	Negative
CFSAN007827	I	Positive	Brown smooth	0.106	Negative
CFSAN007828	I	Positive	Brown smooth	0.153	Moderate
CFSAN007829	I	Positive	Brown smooth	0.178	Moderate
CFSAN007830	I	Positive	Brown smooth	0.132	Moderate
CFSAN007832	I	Positive	Brown smooth	0.144	Moderate
CFSAN007833	I	Positive	Brown smooth	0.135	Moderate
CFSAN007834	I	Positive	Brown smooth	0.131	Moderate
CFSAN007835	I	Positive	Brown smooth	0.222	Moderate
CFSAN007836	I	Positive	Brown smooth	0.133	Moderate
CFSAN007837	I	Positive	Brown smooth	0.217	Moderate
CFSAN007838	III	Positive	Brown smooth	0.312	High
CFSAN007839	III	Positive	Brown smooth	0.309	High
CFSAN007841	III	Positive	Brown smooth	0.169	Moderate
CFSAN007847	III	Positive	Black smooth	0.295	High
CFSAN007848	II	Positive	Black Crystalline	0.278	High
CFSAN007849	II	Positive	Black smooth	0.226	Moderate
CFSAN007850	II	Positive	Brown crystalline	0.259	High
CFSAN007851	III	Positive	Brown crystalline	0.29	High
CFSAN007852	III	Positive	Brown crystalline	0.354	High
CFSAN007853	III	Positive	Brown crystalline	0.345	High
CFSAN007854	III	Positive	Brown crystalline	0.37	High
CFSAN007855	III	Positive	Brown crystalline	0.246	High
CFSAN007856	III	Positive	Brown crystalline	0.277	High
CFSAN007857	III	Positive	Brown crystalline	0.147	Moderate
CFSAN007858	III	Positive	Brown crystalline	0.36	High
CFSAN007859	III	Positive	Brown crystalline	0.359	High
CFSAN007860	III	Positive	Brown crystalline	0.259	High
CFSAN007861	III	Positive	Brown crystalline	0.3	High
CFSAN007862	III	Positive	Brown crystalline	0.361	High
CFSAN007863	III	Positive	Brown crystalline	0.251	High
CFSAN007867	III	Positive	Brown crystalline	0.287	High
CFSAN007868	III	Positive	Brown crystalline	0.352	High
CFSAN007869	III	Positive	Brown crystalline	0.347	High
CFSAN007870	III	Positive	Brown crystalline	0.314	High
CFSAN007872	III	Positive	Brown crystalline	0.32	High
CFSAN007873	III	Positive	Brown crystalline	0.298	High
CFSAN007874	III	Positive	Brown crystalline	0.277	High
CFSAN0078754	III	Positive	Brown crystalline	0.319	High
CFSAN007876	III	Positive	Brown crystalline	0.324	High
CFSAN007877	III	Positive	Brown crystalline	0.345	High
CFSAN007878	III	Positive	Brown crystalline	0.359	High
CFSAN007879	III	Positive	Brown crystalline	0.313	High
CFSAN007880	III	Positive	Brown crystalline	0.273	High
CFSAN007881	III	Positive	Brown crystalline	0.37	High
CFSAN007882	III	Positive	Brown crystalline	0.305	High
CFSAN007883	I	Positive	Brown smooth	0.181	High
CFSAN007884	II	Positive	Brown smooth	0.144	Moderate
CFSAN007886	II	Positive	Brown smooth	0.166	Moderate
CFSAN007887	III	Positive	Brown crystalline	0.389	High
CFSAN007888	III	Positive	Brown crystalline	0.363	High
CFSAN007889	III	Positive	Brown crystalline	0.36	High
CFSAN007892	III	Positive	Brown crystalline	0.285	High
CFSAN007893	III	Positive	Brown crystalline	0.333	High
CFSAN007894	II	Positive	Brown smooth	0.294	High
CFSAN007895	II	Positive	Brown crystalline	0.334	High
CFSAN007896	III	Positive	Brown smooth	0.186	Moderate
CFSAN007897	I	Positive	Brown smooth	0.239	Moderate
CFSAN007898	I	Positive	Brown smooth	0.183	Moderate
CFSAN007899	I	Positive	Brown smooth	0.258	High
CFSAN007901	I	Positive	Brown smooth	0.127	Moderate
CFSAN007902	I	Positive	Brown smooth	0.202	Moderate
CFSAN007903	I	Positive	Brown smooth	0.171	Moderate

*Biofilm classification <0.120 Negative, 0.120–0.240 Moderate, >0.240 High.*

## Discussion

*Staphylococcus aureus* has an immense role in human disease. This investigation studied virulence determinants and the evolutionary relationships for 69 outbreak-linked *S. aureus* isolates using WGS data and *in vitro* phenotypic assessments. Central to the role in *S. aureus* pathogenesis, *agr* is characterized in four allelic groups numbered I–IV. The *agr* locus influences the expression of many *S. aureus* virulence factors (Robinson et al., 2005). This includes roles in regulating genes for biofilm formation and antimicrobial resistance (Tan et al., 2018). The association between biofilm formation and the *agr* groups has been extensively examined. For example, strains of *agr* groups II and III are reported as the main biofilm producers of the four *agr* groups I–IV (Tan et al., 2018). The association among *agr* groups and antibiotic resistance was reported in a study by Nichol et al. (2011) which found a significant association of *agr* group I and community-acquired methicillin-resistant *Staphylococcus aureus* (MRSA), and correlated *agr* group II with hospital acquired MRSA. The *agr* group III was the most prevalent in our analysis, followed by *agr* group I, *agr* group II, and *agr* group IV, which was not found in the isolates tested. Additionally, antibiotic resistance indicator genes *mecA* and *mecC* (Paterson et al., 2014) were not distinguished in our analysis. In a study of mainly clinical isolates of *S. aureus* published by Jarraud et al. (2002), the four *agr* groups were found in relatively even distribution. Jarraud et al. (2002) documented that the *agr* group distribution robustly correlated with the genetic background of the strains tested, and consequently also indirectly with certain disease profiles.

All SE designations have been proven to induce emesis, and numerous outbreak incidences have been reported involving these non-classical SEs. This signifies the need for routine testing to include SEs beyond the classical SEA–SEE. Our analysis identified all SE and SE-like gene targets. Here, we identified six isolates — CFSAN007884, CFSAN007886, CFSAN007896, CFSAN007901, CFSAN007902, and CFSAN007903 — that lacked classical enterotoxins but harbored one or more of the non-classical or SEI gene *seg*–*selx*. This accounts for 9% of the total number of *S. aureus* isolates evaluated in this study. Other examples include a mass SFP outbreak in Osaka, Japan, where greater than 10,000 cases were linked to the consumption of contaminated reconstituted milk due to the presence of small quantities of nearly equal amounts of both SEA and SEH toxins (Ikeda et al., 2005). A different outbreak involving contaminated milk in Romania sickened 30 children. In this outbreak, the *S. aureus* isolates recovered from milk, patients, and food handlers lacked classical enterotoxins A–E and exhibited the same enterotoxin gene profile of *seg*, *seh*, *sei*, and *sem* (Coldea et al., 2015).

Fisher et al. (2018) reported that of the *S. aureus* isolates found in clinical, SFP outbreaks and commensal environments, approximately 80% carry an average of five to six SE genes. Using sequencing analysis, we characterized as many as 11 SEs and SEI genes in several of the *S. aureus* isolates. Both *seb* and *selx* were identified in greater than 50% of the isolates at 54 and 57%, respectively. The enterotoxin genes pattern *sea*, *seb*, *seh*, *sek*, *seq*, and *selx* was the most prevalent among the isolates in this study, while *see*, *ses*, and *set* genes were not detected at all. The toxic shock syndrome toxin gene was identified in 12% and *pvl* was distinguished in 58% for the isolates. These outcomes were compared to previously studied PCR and VIDAS^®^ SET2 results. Results for the functional assays, an end point PCR assay, and WGS were largely in agreement with the exception of new variants of SE*l*U2, SE*l*V, and SE*l*X that were not detected with original primers. The incongruence of SE*l*U is presumably due to gene similarity with SE*l*U2. SE gene concurrence was observed at 96.2%, based on PCR and WGS results.

The determined genes associated with biofilm formation composed of *bap*, *icaA*, and *icaD*, and the genes that encode MSCRAMMs *bbp*, *cna*, *ebpS*, *eno*, *fib*, *fnbA*, *fnbB*, *clfA*, and *clfB* genes. WGS assessment identified the presence of *icaA*, *icaB*, *icaC*, and *icaD* genes in all outbreak isolates. Cucarella et al. (2001) found that all the staphylococcal isolates harboring *bap* were pathogenic and highly adherent biofilm producers. Bap is a surface protein involved in biofilm formation. Our sequencing analyses determined that all isolates were negative for *bap S. aureus* gene V329. However, biofilm-associated genes: *clfA*, *clfB*, *el1S*, *bb1*, *fnbA*, *fnbB*, and *eno* were all present in 99% to all of the isolates examined. Here, *bap* presence was not found to be correlated with expected virulence or biofilm formation ability.

The two methods most commonly described for biofilm detection are the microtiter plate and CRA. The microtiter plate method was developed to replace the first macroscopic method estimation utilizing the tube method. The microtiter plate assay produces qualitative results using a spectrophotometer to measure OD of the 96-well plate containing the stained biofilms. The CRA analysis uses direct colony observation for the characterization of non-slime-producing strains yielding red-colored colonies versus slime-producing strains resulting in black-colored colonies. Here, CRA spot inoculation yielded 52.2% positive results and 47.8% intermediate outcomes, and weak to non-biofilm producers were not identified. The microtiter plate assay distinguished 4.3% of the isolates as weak to no biofilm formation, 36.2% moderate, and 59.4% high biofilm formation among the isolates examined. FDA4-designated isolates were collected during all three inspection dates, with 96% of these isolates being characterized as high biofilm producers. These FDA4 grouped isolates accounted for approximately 40% of the *S. aureus* contamination detected during this investigation. These collated outcomes establish that a significant level of high biofilm-producing strains of *S. aureus* likely assisted in the contamination difficulties found in this bakery environment, particularly given the increased resistance to antimicrobial agents and disinfectants that are characteristic of microorganisms proliferating within a biofilm. Biofilms aid the ability of *S. aureus* to survive in extreme environments.

In addition, the WGS was performed on all isolates from which phylogenetic analysis and genomic examination for virulence factors and biofilm-associated genes were conducted. Importantly, with the detailed metadata information and addition of reference genomes, our sequencing data identified six well-supported distinct clades consisting of the majority of outbreak-related isolates and demonstrated the presence of resident pathogens and multiple introductions in the same facility.

## Conclusion

The evidence indicates that a considerable amount of diversity was observed across the contamination events, which is explained by a combination of resident pathogens with clonal structure and multiple independent introductions. This investigation emphasizes the complexities of eradicating potentially lethal pathogens from food production facilities.

## Data Availability Statement

The datasets presented in this study can be found in online repositories. The names of the repository/repositories and accession number(s) can be found in the article/Supplementary Material.

## Author Contributions

JH and ST contributed to the study design and concept. JH, ST, GC, GK, and JP contributed to the collection of data. JH, GC, JP, and LY contributed to analysis and interpretation of data. JH contributed to drafting of the manuscript. ST, GC, and JP contributed to critical revision. All authors contributed to the manuscript and approved the submitted version.

## Conflict of Interest

The authors declare that the research was conducted in the absence of any commercial or financial relationships that could be construed as a potential conflict of interest.

## Footnotes

1 https://www.ncbi.nlm.nih.gov/pathogens/
