# Supplementary material for: Evaluation of Virulence Determinants Using Whole-Genome Sequencing and Phenotypic Biofilm Analysis of Outbreak-Linked Staphylococcus aureus Isolates
Source: Front Microbiol. 2021 Jun 29;12:687625. doi: 10.3389/fmicb.2021.687625 (PMC8328053; doi:10.3389/fmicb.2021.687625)
Supplement: Supplementary file 1 [file Table_1.pdf]

Table 1. GenBank accession numbers for the 69 Staphylococcus *aureus* isolates

Strain Identifier	Source*	Year	GenBank Accession
CFSAN007820	Environmental Swab	2011	DACXVD010000000
CFSAN007821	Environmental Swab	2011	DACXWL010000000
CFSAN007822	Environmental Swab	2011	DACXVG010000000
CFSAN007823	Environmental Swab	2011	DACXVC010000000
CFSAN007824	Environmental Swab	2010	DACYBA010000000
CFSAN007825	Environmental Swab	2010	DACXVS000000000
CFSAN007826	Environmental Swab	2010	DACXWH010000000
CFSAN007827	Environmental Swab	2010	DACXXL010000000
CFSAN007828	Environmental Swab	2010	DACXVH010000000
CFSAN007829	Environmental Swab	2010	DACXUZ010000000
CFSAN007830	Environmental Swab	2010	DACXUY010000000
CFSAN007832	Environmental Swab	2010	DACXUW010000000
CFSAN007833	Environmental Swab	2010	DACXVE010000000
CFSAN007834	Environmental Swab	2010	DACXWR010000000
CFSAN007836	Environmental Swab	2010	DACXUX010000000
CFSAN007837	Environmental Swab	2010	DACXVY010000000
CFSAN007838	Environmental Swab	2011	DACXVB010000000
CFSAN007839	Environmental Swab	2010	DACXWF010000000
CFSAN007841	Environmental Swab	2011	DACXVV010000000
CFSAN007848	Environmental Swab	2010	DACXVJ010000000
CFSAN007849	Environmental Swab	2010	DACXWJ010000000
CFSAN007852	Environmental Swab	2011	DACXXM010000000
CFSAN007853	Environmental Swab	2011	DACXWT010000000
CFSAN007854	Environmental Swab	2011	DACXWK010000000
CFSAN007855	Environmental Swab	2011	DACXVU010000000
CFSAN007856	Environmental Swab	2011	DACXWM010000000
CFSAN007857	Environmental Swab	2011	DACXWV010000000
CFSAN007858	Environmental Swab	2011	DACXVM010000000
CFSAN007859	Environmental Swab	2011	DACXVK010000000
CFSAN007860	Environmental Swab	2011	DACXVO010000000
CFSAN007861	Environmental Swab	2011	DACXUV010000000
CFSAN007862	Environmental Swab	2011	DACXWG010000000
CFSAN007863	Environmental Swab	2011	DACXWO010000000
CFSAN007867	Environmental Swab	2010	DACYFK010000000
CFSAN007868	Environmental Swab	2010	DACXWQ010000000
CFSAN007869	Environmental Swab	2010	DACXWW010000000
CFSAN007870	Environmental Swab	2010	DACXZS010000000
CFSAN007872	Environmental Swab	2010	DACXWP010000000
CFSAN007873	Environmental Swab	2010	DACXWE010000000
CFSAN007874	Environmental Swab	2010	DACXWA010000000
CFSAN007875	Environmental Swab	2010	DACXXW010000000
CFSAN007876	Environmental Swab	2010	DACXVP010000000
CFSAN007877	Environmental Swab	2010	DACXVR010000000
CFSAN007878	Environmental Swab	2010	DACXWD010000000

CFSAN007879	Environmental Swab	2010	DACXVQ010000000
CFSAN007880	Environmental Swab	2010	DACXVF010000000
CFSAN007881	Environmental Swab	2010	DACXWI010000000
CFSAN007882	Environmental Swab	2010	DACXWU010000000
CFSAN007884	Environmental Swab	2011	DACXVZ010000000
CFSAN007886	Environmental Swab	2011	DACXWN010000000
CFSAN007887	Environmental Swab	2011	DACYAZ010000000
CFSAN007888	Environmental Swab	2011	DACXXV010000000
CFSAN007889	Environmental Swab	2011	DACXVI010000000
CFSAN007892	Environmental Swab	2011	DACXVN010000000
CFSAN007893	Environmental Swab	2011	DACXVX010000000
CFSAN007895	Environmental Swab	2011	DACXWB010000000
CFSAN007897	Environmental Swab	2011	DACXWC010000000
CFSAN007898	Environmental Swab	2011	DACXVT010000000
CFSAN007899	Environmental Swab	2011	DACXVL010000000
CFSAN007901	Environmental Swab	2011	DACXWS010000000
CFSAN007902	Environmental Swab	2011	DACXVA010000000
CFSAN007903	Environmental Swab	2011	DACXVW010000000
CFSAN007835	Environmental Swab	2010	CP017685
CFSAN007847	Environmental Swab	2011	CP017684
CFSAN007850	Environmental Swab	2010	CP017682
CFSAN007850	Environmental Swab	2010	CP017683
CFSAN007851	Environmental Swab	2011	CP017680
CFSAN007851	Environmental Swab	2011	CP017681
CFSAN007883	Environmental Swab	2010	CP017679
CFSAN007894	Environmental Swab	2011	CP017677
CFSAN007894	Environmental Swab	2011	CP017678

* All strains were isolated from IL, U.S.
